# Hand usage and inter-limb coordination parameters for the bimanual rapid visuomotor task to quantify sensorimotor dysfunction of participants with stroke

**DOI:** 10.1186/1471-2202-14-S1-P350

**Published:** 2013-07-08

**Authors:** Kathrin Tyryshkin, Sean P Dukelow, Janice I Glasgow, Stephen H Scott

**Affiliations:** 1 School of Computing, Queen's University, Kingston, ON, K7L 3N6, Canada; 2 Hotchkiss Brain Institute, University of Calgary, Calgary, AB, T2N 4N1, Canada; 3 Centre for Neuroscience Studies, Queen's University, Kingston, ON, K7L 3N6, Canada

## 

Clinical assessment provides a foundation for all aspects of patient care, assisting diagnosis, prognosis and overall patient care. Stroke can impact a broad range of sensory, motor and cognitive functions, but existing assessment tools tend to be largely subjective in nature and use relatively course rating systems. Thus, in practice the majority of stroke patients follow the same general rehabilitation program, which may not necessarily be optimal for each individual case.

The major goal of this research is to develop advanced technologies that provide objective and accurate measurements of stroke-caused impairments and aid clinicians in the planning of individual rehabilitation therapy for stroke patients. The key technology in this research is a KINARM (Kinesiological Instrument for Normal and Altered Reaching Movements) robot that allows for the collection of quantitative measurements of upper limb movements of a subject performing a particular task [1]. The apparatus permits planar movements of the upper limb in concert with a 2D virtual reality system that displays hand position and targets in the horizontal workspace.

This abstract presents a technique for the evaluation of stroke impairment using statistical and time series analysis of the data collected with the KINARM robot. The data were collected from 154 stroke (91 left- and 63 right-affected) and 262 control participants. Each subject completed a bimanual object hit task. In this task participants are instructed to use their right and left hands (represented as green paddles) to hit red balls that move towards the participant. At the beginning of the task, only one ball is displayed and moves relatively slowly, but the number and speed of the balls progressively increases. In addition to completing this object hit task, each stroke participant underwent a standardized clinical stroke assessment.

The data were analyzed and different parameters were extracted that characterize task performance, hand usage motion and ability to multitask. Some of the collected data, such as hand trajectories and hand speed, was represented as time series. Time series and special statistical analyses were performed to develop parameters that quantify whether a specific stroke participant is significantly different from healthy controls. The parameters, taken individually, identified between 14% - 67% of right-side affected and 24% - 88% of the left-affected stroke participants as different from controls. In addition, the parameters allowed to differentiate between spatial and motor deficits in stroke participants. Patients with visuospatial neglect (n = 34) showed greater deficits for some parameters then other stroke participants. Finally, the parameters had significant correlations with collected clinical scores (Functional Independence Measure (FIM), Montreal Cognitive Assessment (MoCA) and Behavioural Inattention Test (BIT)).

## Conclusions

The object hit task and the developed parameters quantify participant performance including overall task success, spatial and temporal aspects of behaviour, and the motor abilities of each arm. The technique effectively detects abnormalities in the performance of stroke patients in planning and control of goal-directed movements.
